# Exploring the role of a facilitator in supporting family carers when embedding the iSupport for Dementia programme in care services: A qualitative study

**DOI:** 10.1111/jocn.16836

**Published:** 2023-07-21

**Authors:** Ying Yu, Sarah C. Hunter, Lily Xiao, Claudia Meyer, Michael Chapman, Kai Ping Tan, Langduo Chen, Sue McKechnie, Julie Ratcliffe, Shahid Ullah, Alison Kitson, Andre Q. Andrade, Craig Whitehead

**Affiliations:** ^1^ College of Nursing and Health Sciences Flinders University Bedford Park South Australia Australia; ^2^ Caring Future Institutes Flinders University Bedford Park South Australia Australia; ^3^ Bolton Clarke Research Institute Melbourne Victoria Australia; ^4^ Rehabilitation, Ageing and Independent Living Research Centre Monash University Clayton Victoria Australia; ^5^ Centre for Health Communication and Participation La Trobe University Melbourne Victoria Australia; ^6^ Australian National University Canberra Australian Capital Territory Australia; ^7^ Department of Palliative Care Canberra Health Services Garran Australian Capital Territory Australia; ^8^ Southern Adelaide Local Health Network Adelaide South Australia Australia; ^9^ Community Services Resthaven Incorporated Bartley Crescent Wayville South Australia Australia; ^10^ College of Medicine and Public Health Flinders University Bedford Park South Australia Australia; ^11^ Clinical and Medical Sciences, Quality Use of Medicines and Pharmacy Research Centre University of South Australia Adelaide South Australia Australia

**Keywords:** caregiver support, co‐design, dementia, dementia care services, facilitator, family caregiver

## Abstract

**Aims:**

To explore stakeholders' perceptions of a facilitator's role in supporting carers when embedding iSupport for Dementia psychoeducation program, in care services.

**Methods:**

A qualitative descriptive study design was applied. Data were collected from workshops and interviews with carers of people living with dementia (PLWD)and with health and social care professionals from two tertiary hospitals and two community aged care organisations across three Australian states between October 2021 and March 2022. A thematic analysis was used to analyse data. The COREQ guideline was followed to report our findings.

**Results:**

A total of 30 family carers and 45 health and social care professionals participated in the study. Three main themes and seven subthemes were identified from the data. We described the main themes as (1) the facilitator's role at the time of dementia diagnosis, (2) the facilitator's role throughout the everyday dementia care journey and (3) the facilitator's role during transition moments.

**Conclusions:**

Caring for family members with dementia is demanding and stressful for carers. Embedding a facilitator‐enabled iSupport for Dementia program in hospital and community aged care settings has the potential to mitigate sources of stress associated with care recipient factors, carer factors and care service factors, and improve the health and well‐being of carers and those for whom they care.

**Relevance to Clinical Practice:**

Our findings will inform the establishment of iSupport facilitators appointed by dementia care providers in hospital and community care settings and help determine their roles and responsibilities in delivering the iSupport program. Our findings relate to nurse‐led and coordinated dementia care in hospital and community aged care settings.

**Patient or Public Contribution:**

This study was co‐designed with stakeholders from two aged care organisations and two tertiary hospitals. The study participants were staff employed by these organisations and carers of PLWD who were service users.

**What does this paper contribute to the wider global community?**

- Embedding iSupport into dementia care services in hospital and community aged care settings is a way to enhance dementia care education and improve the quality of home‐based care for people with dementia.
- Implementing the facilitator‐enabled iSupport for Dementia program has the potential to mitigate sources of carer stress to which care recipient factors, carer factors and care service factors contribute.

## 1 INTRODUCTION

Worldwide, there are 55 million people living with dementia (PLWD), and most of them are cared for by family carers (carers hereafter) at home (World Health Organization, 2021). PLWD have higher rates of health and social care services usage than those without dementia due to complex care needs arising from dementia and multimorbidity (Anderson et al., 2020; Bott et al., 2019). They also rely on their family carers to care for them and act on their behalf to communicate their care needs to service providers. Nurses in hospital and community aged care settings play a leading role in managing and coordinating care for PLWD, including working with carers to keep PLWD living at home for as long as they wish. However, their role in supporting carers in managing dementia in home care settings remains largely unclear as carers are not viewed as clients in current health and social care systems. The lack of support for carers limits opportunities for them to obtain ongoing dementia care education, participate in care planning with care service providers and access and use care services to meet the care needs of PLWD. Studies have revealed that enhancing support for carers could significantly reduce complications, prevent both hospital admissions and premature admissions to permanent nursing home care for PLWD and result in cost savings for health and social care systems (Anderson et al., 2020; Bott et al., 2019).

To strengthen support for carers, the World Health Organization (WHO) has developed an iSupport for Dementia (iSupport hereafter) program, a comprehensive psychoeducation program for carers aimed at improving their ability to manage dementia at home and reduce their stress (World Health Organization, 2021). The iSupport program has been culturally adapted for Australians as an online and paper‐based resource for carers (Xiao et al., 2021). The Australian iSupport program comprises six learning modules: (1) Introduction to Dementia, (2) Being a Carer, (3) Caring for Me, (4) Providing Everyday Care, (5) Dealing with Changed Behaviour and (6) My Engagement in Consumer‐Directed Care. Dementia care education and training for carers have mainly been provided by Alzheimer's associations to date in many countries, including Australia. However, studies show that carers expect one‐stop‐shop services provided by skilled health and social care professionals where the PLWD receives care services (Steiner et al., 2020). Studies of the iSupport program in Australia revealed that carers expected to have a designated iSupport facilitator to bridge gaps in health and social care systems (Xiao et al., 2021; Xiao, Ye, et al., 2022). Further work to explore the functions of this facilitator role in embedding iSupport into hospital and community aged care services was needed. This study responds to the need for consumer‐driven dementia care services by co‐designing the iSupport facilitator's role with stakeholders. In this study, the ‘iSupport facilitator’ is defined as a trained health or social care professional appointed by health or social care organisations to enhance support for carers of PLWD by embedding iSupport in their routine care services. This study is part of a large project entitled ‘Creating “Partnership in iSupport program” to optimise family carers' impact on dementia care’ published elsewhere (Xiao, Yu, et al., 2022). The project includes three phases: (1) Phase 1: co‐design activities delivered by an iSupport programme facilitator working with stakeholders; (2) Phase 2: assess the intervention's effectiveness and cost‐effectiveness and understand carers' experiences in the programme; and (3) Phase 3: translate the ‘Partnership in iSupport program’ into practice. This study reports the main findings from Phase 1 of the project.

## 2 BACKGROUND

Research evidence consistently demonstrates that carer stress is associated with PLWD factors. PLWD are often highly dependent on carers to maintain activities of daily living (i.e. showering, toileting) and instrumental activities of daily living (i.e. paying bills, transportation) compared to those without dementia. Many carers spend over 35 h per week assisting PLWD through care activities, which significantly limits their opportunities to socialise with others (Farina et al., 2017). Many PLWD also have up to five chronic conditions that require carer management on a daily basis (Australian Institute of Health and Welfare, 2021). Furthermore, most PLWD manifest changed behaviours, such as confusion, agitation and sleep disturbances, that add to the physical and psychological stress of carers and affect the relationships of the carer–care recipient dyads (Chunga et al., 2021; Feast et al., 2016). Carers of PLWD who have more significant episodes of changed behaviour have higher levels of daily distress. Without intervention, these care‐recipient‐related stressors will have adverse consequences on carers' health and well‐being.

Factors associated with the carers themselves also contribute to carer stress. A recent global survey by WHO identified that only a small proportion of countries provided carers with full access to dementia education and training (World Health Organization, 2021). Inadequate dementia care education for carers is associated with inability to manage dementia at home and increased caregiver burden (Allen et al., 2020). Embedding iSupport in hospitals and community aged care organisations is an opportunity to enable carers to access dementia education when they need it. Moreover, acquiring knowledge from existing education programmes is insufficient for carers to cope with everyday challenges. Studies have revealed that carers need to develop self‐efficacy (i.e. a sense of competence) in order to efficiently obtain respite, deal with care recipients' changed behaviour and control negative thoughts such as perceptions of loss of self and role captivity (Pearlin et al., 1990). According to Bandura's (1993) cognitive development theory, developing self‐efficacy requires carers to learn from a peer role model, be motivated by others (facilitators and peers) in goal setting, gain emotional support from others when needed and take action to translate knowledge into daily care practice. Studies also reveal that family dynamics may impact the support carers gain from other family members (Smith et al., 2022). Furthermore, carers who are still in the workforce may experience role strain when there are conflicts between their jobs and their caregiving roles. Prolonged care stress is associated with negative thoughts in carers and can contribute to mental health issues such as anxiety and depression (Alcindor, 2022). Carers' positive thoughts (i.e. perceptions of a sense of achievement and personal gain), however, enable them to reduce stress and maintain mental health (Yu et al., 2018). Carers' positive thinking can also be enhanced by sharing and confirmation during interactions with peers.

Similar to other developed countries, Australia possesses various community aged care services to support PLWD to live at home for as long as they wish, and these services are subsidised and regulated by the Australian Government (Department of Health and Aged Care, 2022). Aged care services (i.e. transport, food, housework, and personal care) are provided at an entry level through the Commonwealth Home Support Programme and at advanced levels such as the Home Care Package (Level 1 to Level 4). The Australian Government also funds short‐term care such as transition care (up to 12 weeks), restorative care (up to 8 weeks) and respite care. Hospitals are usually involved in referring PLWD to these care services to shorten hospital stays. To access government‐funded services, people must apply for an assessment through the website ‘My Aged Care’, and waiting periods vary. A consumer‐directed care model empowers users to control the allocated budget, choose service providers and negotiate services to meet their care needs. However, carers view the aged care services as highly fragmented and difficult to access and use when needed (Steiner et al., 2020). Long waiting periods and inflexible respite care were reported and viewed as unacceptable by carers (Steiner et al., 2020). In addition, poor‐quality care services, for example, miscommunication between service providers and a lack of continuity of care, were reported by carers (Tuijt et al., 2021). Therefore, carers wish for a link worker who acts as a single point of contact for a minimum of 1 year postdiagnosis to provide support in the health and social care systems (Goeman et al., 2016).

Studies on dementia care providers' roles in supporting carers in hospital and community aged care settings where PLWD receive care services are rare. This study addresses this gap in research by co‐designing an iSupport facilitator's role with stakeholders to strengthen support for carers in two tertiary hospitals and two large community aged care organisations, all of which planned to embed the iSupport program into their care services. The aim of the study was to explore stakeholders' perceptions of facilitators' roles in supporting carers when embedding iSupport into care services in hospitals and community aged care organisations.

### 2.1 A conceptual framework

**FIGURE 1:**
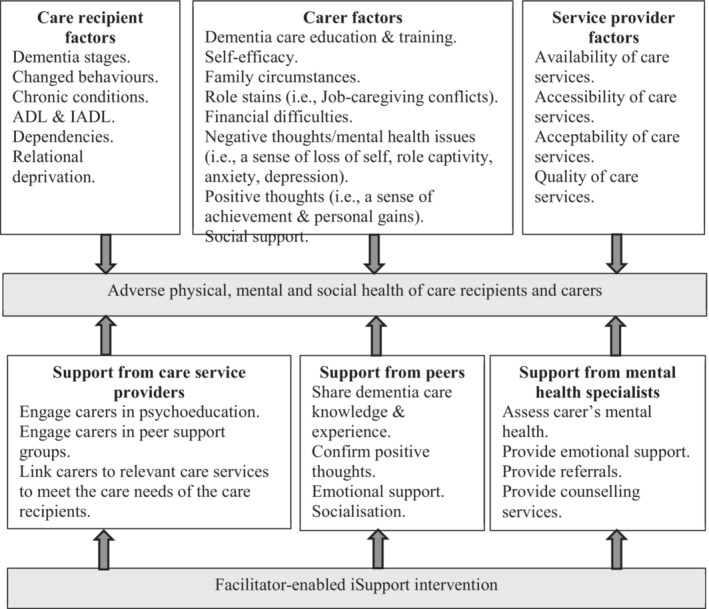
A facilitator‐enabled iSupport intervention framework to strengthen support for carers.

Based on the aim of the study, a literature review and an analysis of previous conceptual models, we propose a facilitator‐enabled iSupport intervention framework to strengthen support for carers to inform the study design (Figure 1). Our framework takes into account the ‘Stress and Health Process’ model developed by Pearlin et al. (1990), who categorised the sources of carer stress into four domains: (1) background and context (i.e. carer's socio‐demographic factors, access to networks and social support, and availability of programmes), (2) primary stressors (i.e. the PLWD factors: dependence level, changed behaviour and other comorbidities), (3) secondary role strains (i.e. family conflicts, constrictions of social lives, work–caregiving conflicts and financial problems) and (4) intrapsychic strains (i.e., negative thoughts: perceptions of loss of self and role captivity). We describe the primary stressors in Pearlin's model as ‘care recipient factors’ to alert care service providers to assess these sources of stress. Moreover, we combine the stressors associated with carers' background and context, secondary role strains and intrapsychic strains into a single category, ‘carer factors’, to enable care service providers to focus on carers, the main stressors they face and the relationships between these stressors; for example, a lack of dementia education may also contribute to negative thoughts during dementia care. Thus, the care service intervention needs to be designed to target each of those sources of stress simultaneously when appropriate (Figure 1). Pearlin's model did not include service provider factors, which have been widely reported in the literature as sources of stress for carers (Steiner et al., 2020). Therefore, we have added ‘service provider factors’ to our framework and emphasised that carers' concerns about the availability, accessibility, acceptability and quality of dementia care services are sources of stress for them.

Our framework also takes into account the suggestion by Conde‐Sala et al. (2010) that interventions to improve the health and well‐being of carers need to include three types of support: social support (educational, emotional and family associations), social resources (domiciliary, community and institutional care) and treatments (pharmacological treatments, cognitive stimulation and psychotherapy). Based on our study's aim, we reframe these three types of intervention as (1) ‘support from care service providers’, to emphasise their role in engaging carers in dementia care education and peer support and in linking them to relevant care services to meet the care needs of PLWD; (2) ‘support from peers’, to address socialisation with others; and (3) ‘support from mental health specialists’, to emphasise the need to assess carers' mental health and provide referrals accordingly (Figure 1).

## 3 METHOD

### 3.1 Study design

The qualitative descriptive approach described by Doyle et al. (2019) was applied to interpret participants' perceptions of the iSupport facilitator's role in supporting carers of PLWD. This approach enables researchers to interact with participants to elicit their perspectives on a topic of interest based on their experiences. This approach also allows researchers to interpret findings that are very similar to participants' viewpoints. We followed the Consolidated Criteria for Reporting Qualitative Research checklist (Data S1) for reporting qualitative studies (Tong et al., 2007).

### 3.2 Settings and participants

This study was conducted in two tertiary hospitals and two large aged care organisations across three of Australia's states: South Australia, Victoria and Australian Capital Territory (ACT) between October 2022 and March 2023. Ethics approval was obtained from the Southern Adelaide Clinical Human Research Ethics Committee (No. 2021/HRE00273) and Bolton Clarke Human Research Ethics Committee (No. 21007). Written informed consent was obtained from all participants. Participants in this study included two cohorts: (1) carers of PLWD who were clients of the participating organisations and (2) health and social care professionals of the participating organisations. Participants were recruited using convenience and snowball sampling strategies. Carer participants were offered a gift card to compensate them for the time they spent on the study. Health and social care professional staff participants participated in the study during their paid working hours.

Carers of PLWD were invited to participate if they were aged 18 years or over, provided care activities at least three times a week and had cared for a PLWD for at least 6 months. Health and social care professionals from various disciplines, for example, nurses, medical doctors, physiotherapists, occupational therapists and community aged care coordinators, were invited to participate if they had at least 1 year of experience in dementia care. Potential carer participants were identified by clinicians or volunteered after seeing an advertising flyer in one of the organisations. Potential staff participants were informed of the study through information sessions presented by the researchers during various staff meetings or via flyers displayed in staff rooms. One of the researchers contacted potential participants based on their expressions of interest, assessed their eligibility and obtained their written consent.

### 3.3 Data collection

We applied the modified co‐design data collection method described by Goeman et al. (2019), which included iterative consultations with stakeholders to ensure that the key aspects of the iSupport facilitator's role would be adequately delineated, acceptable to stakeholders and implementable in real care settings. The project team developed a draft definition of the iSupport programme facilitator's role to engage participants in discussions (Table 1) based on our previous studies of stakeholders' expectations of iSupport implementation in Australia (Xiao et al., 2021; Xiao, Ye, et al., 2022) and a systematic review on the effectiveness of the support worker role for carers of PLWD (Goeman et al., 2016). We invited 10 associate investigators from the project, who were experts in various disciplines (aged care and dementia care nursing, geriatrics, geropsychiatry, gerontology, psychology and pharmacology, a past carer and a consumer representative) and 12 project advisory group members (five carers, two PLWD at early stages and five professional care staff) to review and revise the listed activities. Differences among group members on the draft list were resolved through online meetings and email communications.

**TABLE 1:** A draft list of the iSupport program facilitator's role to engage participants in discussions.

Items	iSupport program facilitators support carers to:
1	navigate, access and use dementia care resources and multidisciplinary care services to meet the care needs of the person with dementia and their carers.
2	use self‐care strategies to take care of themselves and to reduce stress and distress.
3	prevent and/or manage dementia‐related symptoms and changed behaviours.
4	manage chronic conditions the person with dementia has, (i.e. hypertension, diabetics and other chronic diseases and conditions).
5	identify risks, causes and triggers that will contribute to presentation at the emergency department.
6	identify risks, causes and triggers that will contribute to hospital admission.
7	identify risks, causes and triggers that will contribute to premature permanent admission to residential aged care homes.
8	identify early signs of deterioration in the person with dementia.
9	manage transitions between care settings (i.e. hospital‐to‐home) and care types (i.e. receiving palliative care at home.
10	work with professional staff to develop or revise care plans for home care packages or in a care plan in hospital clinics to meet the needs of the person with dementia.
11	assess their learning needs, and choose relevant learning units from the online iSupport for Dementia program to help them look after themselves as well as the person with dementia.
12	participate in virtual carer support groups to gain social support and to exchange their dementia care knowledge, skills and experiences.
13	provide feedback to service providers regarding the strengths of the care services and the areas that need to be improved.
14	provide a personal touch and a single point of contact with a live person rather than a computer or telephone activated voice.
15	coordinate the virtual carer support groups.
16	What activities do you suggest an iSupport program facilitator could undertake to support carers of people living with dementia?

Prior to workshops and interviews, we invited carers and staff participants to review, rate and comment on the draft definition of the iSupport programme facilitator's role and add new activities, via a preworkshop survey. Participants attended workshops or interviews to discuss their perspectives on the iSupport programme facilitator's role. We also invited participants to complete a postworkshop survey. In this paper, we present findings from the qualitative data generated from the workshop and interviews only. The pre‐ and postworkshop survey findings will be reported elsewhere.

Participants were invited to attend one of the scheduled workshops, or to have a phone interview if they were unable to attend the workshops. Workshop discussions were conducted by two researchers at each study site, while phone interviews were undertaken by a single researcher at each study site. All researchers involved in data collection had training and experience in qualitative research. During the workshop or interview, the researcher introduced the collated preworkshop survey results to participants and facilitated their discussions of the listed iSupport facilitator's roles (Table 1), along with any activities added by participants. The workshop discussions and the interviews were audio‐recorded and transcribed verbatim for data analysis. Each workshop lasted, on average, around 90 min, while the individual interviews lasted around 40 min. Face‐to‐face workshops were conducted in South Australia, where COVID‐19 restrictions were not in place, and participants in those workshops were not required to wear masks. Online workshops were conducted in Victoria and ACT due to COVID‐19 restrictions. Data saturation was achieved, as evidenced by the presentation of repeated information.

### 3.4 Data analysis

The six‐step thematic analysis introduced by Nowell et al. (2017) was used for the data analysis. The first author led the data analysis by (1) familiarising themself with the transcripts; (2) generating initial codes and grouping those codes based on similarities and differences; (3) searching for potential themes by analysing the concepts arising from the grouped codes, the relationships among these concepts, and how the codes related to the study's aim and conceptual framework; (4) reviewing themes with the team; (5) naming themes; and (6) producing the report. A computer‐assisted qualitative data analysis programme, NVivo (released in March 2020), was used to manage data and facilitate coding (QSR International Pty Ltd, 2020). The first author completed the first‐round data coding, and the third author checked and validated that coding. The codes, group codes, preliminary themes and subthemes were reviewed by the project team and then refined to address the team's feedback. We invited the project advisory group members to review and comment on the findings and make changes accordingly.

### 3.5 Study rigour

The trustworthiness of the study was ensured by considering the study's credibility, confirmability, dependability and transferability (Liamputtong, 2019). The research team collectively has expertise in qualitative study design. The study's credibility was enhanced by transcribing the recorded voice data verbatim for data analysis. Moreover, the transcripts were checked by the researchers who conducted the workshops and interviews to ensure the accuracy of the data. In addition, this data accrual was conducted over a prolonged period of engagement with participants, over 6 months, during this phase of the study, which increased the confidence of the findings. The confirmability of the study was strengthened by having team members crosscheck the codes, subthemes and themes and by presenting quotations from participants, when possible, to ensure participants' views were correctly presented and interpreted. Differences in opinion on findings existed among team members but were resolved through discussion. The study's dependability was ensured by providing training to the researchers involved in the data collection and data analysis and by complying with a clearly documented study protocol to minimise disparities in the data collection process between different sites and researchers. Moreover, the same interview questions were used for all participants. The telephone interviews were conducted after the workshops, and a brief summary of the workshop discussions were provided to the telephone interview participants to prompt their responses and ensure their views were fully captured. All researchers met regularly during the data collection and data analysis periods to ensure the defined method was used. The transferability of the study was demonstrated by a detailed discussion of the findings and the context of the study.

## 4 RESULTS

In total, 30 carers of PLWD and 45 health or social care professionals (*n* = 75) participated in one of 14 scheduled workshops or interviews. The carer cohort comprised spouses (*n* = 23), adult children (*n* = 5), siblings (*n* = 1) and grandchildren (*n* = 1). The health and social care professionals were from various disciplines; they included nurses (*n* = 20), aged care coordinators and managers (*n* = 14), physiotherapists (*n* = 4), medical doctors (*n* = 3), occupational therapists (*n* = 3) and social workers (*n* = 1). The demographic characteristics of the participants are presented in Table 2.

**TABLE 2:** Demographic characteristics of participants (*n* = 75).

Participants characteristics	Staff *n* (%)	Family carers *n* (%)
Number of participants who provided demographic information *n* (%)	36 (65.45)	19 (34.55)
Age: mean (SD)	46 (11.4)	73 (11.55)
Gender *n* (%)		
Male	3 (8.6)	1 (5.3)
Female	32 (91.4)	14 (73.7)
Years in the service: mean (SD)	15.83 (9.72)	N/A
Years in the carer role: mean (SD)	N/A	5.88 (3.63)
Relationship with the care recipient	N/A	Adult Child = 1 Grandchild = 1 Spouse =13
Occupational categories	Registered Nurse =11 Enrolled nurse = 1 Social care professionals = 8 Physiotherapist = 5 Occupational therapist =3 Geriatrician =1 Medical officer = 1 Social worker = 1	Employed =1 Unemployed =2 Retired = 15

*Note*: (1) Social care professionals including care advisor, case manager and care coordinator in community aged care. (2) Please note that 20 out of 75 participants did not provide their demographic information via the online survey prior to the workshops. (3) Some participants did not answer all demographic question.

Three main themes, with seven subthemes, were identified during the data analysis. We described the main themes as (1) the facilitator's role at the time of dementia diagnosis, (2) the facilitator's role throughout the everyday dementia care journey and (3) the facilitator's role during transition moments. In this article, we use ‘IN’ to indicate quotations from interview data and ‘G' to indicate data from group discussions in workshops. Group 10 included both carers and health and social care professionals. Therefore, the quotations are noted as G10_carers and G10_staff. All other groups had either carer or health professional participants only.

### 4.1 Theme 1: The facilitator's role at the time of dementia diagnosis

Carer participants perceived that dementia care services and resources were not organised to be easy for them to navigate and access. They also perceived that they had been unaware of where or who to approach for help at the time of dementia diagnosis. Staff participants perceived that they had limited time to assist carers with navigating information and services. Both carers and staff perceived that a designated iSupport facilitator should address unmet needs of carers at the time of dementia diagnosis, as detailed under the following subthemes.

#### 4.1.1 Subtheme 1.1: The need to support carers to navigate dementia care services and resources

At the time of dementia diagnosis, carers were not provided with the breadth of support they needed. For example: ‘Well day one when they tell you that you have got terminal Alzheimer's, does not tell you all the legal, all the basics. Power of Attorney was critical because if you do it later, you get into trouble’ [G1_carer]. Another carer stated: ‘It can be very daunting in the beginning … As far as all the things that are available to you … accessing them and finding out about all the help that you can get’. [IN1_carer]. Carers expected the facilitator to help them at the point of diagnosis: ‘I think that person needs to be there at the time when the diagnosis is made … because it is then that you need the information, not six months later’ [G4_carer].

Carers also encountered difficulties in understanding terms: one carer stated: ‘It takes a while for you to work out what actually an ACAT [aged care assessment team] is and whom you need to see’ [G4_carer]. Working carers perceived role strain from finding the information they needed: ‘I have got to work, I have got to spend time with the kids, I've got to spend time with the family … I just don't know what the resources are and how to access them’ [G14_carer]. This was echoed by another working carer: ‘To have somebody to talk to or guide me or say—just advise me on how, not to be shocked and taken aback every time and think, what is going on? … So that is very much needed’ [G1_carer].

#### 4.1.2 Subtheme 1.2: The need to help carers understand dementia and care services

Staff reported that time constraints limited how much they could support carers: ‘We have very little time to sit with people, and we really need to explain to them what dementia is, what's delirium’ [G7_staff]. Staff also acknowledged that the system is complex for carers to navigate: ‘We end up referring a lot of carers to social workers to assist with even really minor things like getting people set up with Dementia Support Australia’ [IN 2_staff]. The complexity of the dementia and aged care system was also challenging for staff to navigate: ‘It's nearly a full‐time job for me to navigate the system let alone trying to teach a carer’ [G11_staff].

Information overload was another concern: ‘It is overwhelming being presented with that information just once as well. So, people need time to think about it … you gave them all this paperwork and they are like oh it is too hard’ [IN2_staff]. Staff perceived that an iSupport facilitator could be in an ideal position to address the unmet information needs and the need for ongoing support: ‘They need some support as well from someone’ [IN2_staff].

Findings categorised under this theme revealed that the sources of stress carers encountered at the time of diagnosis were attributed to carer factors (inability to access information and social support), role strains for carers in paid employment, and service provider factors (limited time to provide emotional and information support for carers, or lack of support for carers). Participants perceived that a designated iSupport facilitator could mitigate these sources of stress by helping carers access support networks and trustworthy information, helping them plan their care journeys and providing timely emotional support for them.

### 4.2 Theme 2: The facilitator's role throughout the everyday dementia care journey

Participants described that managing changed behaviour and daily medication for PLWD were the main sources of stress for carers. Moreover, carers often experienced emotional and psychological stress and even reached crises, but they rarely sought help from others. They believed that a designated iSupport facilitator could play a crucial role in supporting carers to cope with stress and stabilise home care, as detailed under the following subthemes.

#### 4.2.1 Subtheme 2.1: The need to support carers to manage care recipients' changed behaviour

Carers in this study learnt how to identify causes and triggers of changed behaviour, mainly through a trial‐and‐error approach over time: ‘There was a few triggers with dad that we worked out later on. We did not understand why he was getting so aggressive’ [G4_carer]. Another carer echoed a similar experience: ‘[I] gauge whether it is okay in this situation when I am dealing with the dementia? … it has taken me a while to hopefully get some confidence in myself that I can read the situations now’ [IN1_carer].

Carers would like to have tailored dementia care education to help them to effectively prevent or manage changed behaviour: ‘That is important at the right time, because you have to know what can happen and what alternatives you have before its happens’ [G2_carer]. Carers recommended that a facilitator, as a real person who could provide ongoing support to help them deal with changed behaviour, would be beneficial: ‘I would prefer to access to a person or a workshop, rather than reading a bunch of papers online’ [G14_carer]. Carers perceived that acquiring knowledge and skills in dementia care was a slow process and involved trial and error over time. For example, one carer stated: ‘I have learnt over the years to make my husband as happy as I can, as he wants. Communicate with him in such a way that it does not frustrate him’ [G14_carer].

Staff perceived that carers' behaviour usually triggered the changed behaviour of PLWD:

Because the husband or wife wants them to do the things that they did prior and they've lost enough capacity that they can't do those things, but they want to try and encourage them and that's great, but then the person with dementia is getting really agitated. [G3_staff]

Therefore, staff underscored carers' participation in dementia education: ‘Education and how to manage the best to their ability. You cannot really change the behaviour of the client … the carer can change their behaviour or their look into their parent by obviously understanding that they do have dementia’ [G12_staff]. Staff suggested that ‘a support worker could help them [carers] to identify the fact that they're starting to … You can tell when someone's not coping well even with a phone call’ [G3_ staff]. The suggestion indicates that iSupport facilitators need to be skilled health or social care professionals who usually provide direct care to PLWD.

#### 4.2.2 Subtheme 2.2: The need to support carers to manage medication at home

People with dementia usually depended on their carers to manage their daily medications. Staff strongly suggested the need to provide carers with medication education so that they could advocate for their care recipients: ‘You often see one change in medication, huge change in them [PLWD] going to the toilet, their behaviour, their sleeping patterns. So, I think there needs to be more advocacy around that for them’ [G5_staff]. Furthermore, staff recognised that organising medication review for PLWD in the community could be a challenging task: ‘The problem is, if you're out in the community, and you rock up at a pharmacist for a medication review. They can't really do much unless you go via the GP [general practitioner)’ [G7_staff]. Therefore, staff suggested that a facilitator could smooth the review process [G5_staff].

Carers perceived that information about medication management provided by health professionals was not consistent: ‘There's conflict between when I'm told by someone, when I'm told by someone else’ [G2_carer]. Therefore, they had to ask for help from a trusted community pharmacist: ‘I go to the pharmacist … and they know what you have got and what you haven't got … actually I take the pharmacist's advice more than I take some doctor's advice’ [G2_carer]. Staff also echoed similar concerns about inconsistent information about medication provided to the carers: ‘If you go to one specialist, a heart specialist, it says you do not take this tablet … you go to the kidney specialist, and they say oh, you need it now. They are getting mixed messages’ [G13_staff].

The PLWD usually experienced constant medication changes due to complex health conditions. This situation was a source of stress for carers: ‘The numbers of medication changes, not just doses, but the medication itself, and that was expensive, difficult’ [G4_carer]. Staff suggested that a facilitator could play a crucial role in supporting carers to reduce stress associated with medication changes: ‘If there is actual support there then the likelihood is that their medications are being well‐managed’ [G8_staff]. Moreover, the facilitator could also enable carers to use various methods such as Webster packs to prevent them spilling or mixing up the medicines as ‘some carers do not know there was Webster pack’ [G5_ staff]. However, carers questioned the possibility of facilitators having a role in medication management: ‘I do not know whether a facilitator would help … a facilitator wouldn't know more than the prescribing doctor, I'm sure’ [G4_carer]. The findings indicate that the facilitator needs to be a skilled health professional or play a coordinator's role in assisting carers in communicating with pharmacists, GPs and registered nurses regarding medication management when needed.

#### 4.2.3 Subtheme 2.3: The need to support carers to cope with emotional stress

Carers often experienced emotional stress: ‘As a carer I know I've run the full gamut of all those emotions … that you're in a hopeless cruel situation in so many ways. So, getting that support is vital’ [IN1_carer]. However, they rarely shared their stress with health and social care professionals or asked for help from them: ‘Just somebody else sticking their nose in our business when they offered us mental health support, because we just didn't want to have to keep telling different people the same story over and over again’ [G4_carer]. Staff participants suggested that to modify such a situation, it was important that relationships with carers involved trust: ‘You will need to find some ways to build up trust relationship for us to be able to understand each other and ready to find that to help them’ [IN2_staff]. Carers further described the reason for not seeking help when times were stressful: ‘I do not think anyone else really understands, and especially if they're not going through our journeys. You can sit here and talk to me for as long as you want, but you don't understand’ [G4_carer]. This case emphasised the need for staff and facilitators to demonstrate empathy.

Staff were involved in referring carers to mental health services when they found that carers were in a crisis: ‘There have been a few cases where I've had to get the aged care mental health service involved, to do an urgent assessment and hospitalisation of some of my clients because they've just had such huge dementia crises’ [G12_staff]. In such cases, mental health support came too late and was insufficient. Staff were aware that their ability to provide counselling support for carers was limited: ‘I think we could provide mental health support to a point. And I think then it needs to be specialised’ [G7_ staff]. Carers and staff agreed that a facilitator could ‘offer timely personalised support in person’ [G14_carer], ‘enhance the relationship building' [IN2_staff], ‘better understand carers’ needs' [IN2_staff] and ‘[link] carers to mental health support services’ [G3_staff].

Overall, Theme 2 revealed the sources of stress related to the changed behaviour of PLWD (care recipient factors), difficulties managing dementia and medication (carer factors) and lack of timely emotional support for carers (service provider factors). Participants perceived that a facilitator could play crucial roles as an educator, resource person and coordinator who could support carers in managing changed behaviour and reviewing medication and as a trusted professional who could check carers' mental health status and provide timely interventions and referrals.

### 4.3 Theme 3: The facilitator's role during transition moments

Carers often experienced significantly stressful situations during transition moments, for example, being unable to care for their care recipients when they themselves experienced health issues or when their care recipients experienced changes or transitions between home and hospital. The subthemes detailed in the following subsections indicate the role a designated iSupport facilitator could play during transition moments.

#### 4.3.1 Subtheme 3.1: The need to help carers obtain timely respite care services

Carers usually experienced difficulties in obtaining respite care for PLWD, especially when PLWD presented with changed behaviour: ‘I was on the phone trying to organise a respite before I went for my surgery, but no one would take him [due to changed behaviours]. We were both in the hospital at the end’ [IN4_carer]. This case indicated that a provider factor was those with changed behaviour being excluded from respite care. A lack of suitable respite care for PLWD was also observed by staff who worked in the hospital settings: ‘It's always a known fact that just before Christmas you get a lot more dementia patients so families can go on holiday’ [G3_staff]. Such situations revealed that avoidable hospitalisations for PLWD were due to difficulties in accessing respite care when carers needed it. Some carers reported a lack of competence among staff caring for PLWD in nursing home respite care: ‘Kathy [pseudonym] got expelled from the nursing home after a day because they just could not do anything with her’ [G4_carer]. Long waiting times were also a stressor for carers: ‘Because Veterans Affairs told me you are entitled to so many hours, I thought okay, I am going to put her in somewhere for a couple of days so I can reboot … But then I have got to wait however long' [G1_carer]. Carers often needed to fill out multiple forms and contact multiple agencies: ‘It is overwhelming, and there are some things that I've got sitting here that I'm supposed to do’ [G10_carer]. They often made tireless efforts to access services: ‘You need a lot of time and effort to kind of be able to access exactly what the service provider provides, and it can be multiple service providers as well’ [IN2_staff].

Staff were also concerned that respite care was not accessible for some PLWD: ‘In‐home respite is quite more expensive … you would only really be able to have that if you have got Level 4 [Home Care Package]’ [G5_staff]. Staff also noticed a reluctance to use respite care among some carers due to the guilt they felt: ‘They feel if they are not there to provide care. At least they could be physically there with the patient … it is hard especially when the patient is elderly and the carer is also an elderly person’ [IN2_staff]. Meanwhile, underutilised respite care was also observed by staff: ‘So we do definitely see patients where they're underutilising their Home Care Package, not because it's not available but just because they didn't know they could ask for more help’ [IN2_staff]. Both carers and staff welcomed the idea of a designated facilitator who could ‘link them to available services’ [G3_staff] to alleviate stress because ‘they often do not know where to start’ [G14_carer].

#### 4.3.2 Subtheme 3.2: The need to support carers during the hospitalisation of their care recipients

Hospital admission was a significant source of stress for carers. Carers rarely understood the information provided by healthcare staff during hospitalisation: ‘It's been a nightmare of just, once you're in the system you just get random phone calls from people that tell you things, and you don't have the capacity to internalise it and understand what they're telling you’ [G14_carer]. Carers also experienced stress when they observed a lack of dementia care strategies in the hospital settings:

She was in a room by herself. She had the railings up. But for her to get the railings down to go to the loo, on the floor, which she would not understand … she would have fallen. And because the staff are so busy, and they couldn't be there—they can't be there 24/7. [G9_carer]

Carers' stress increased when they were unable to be the voice of PLWD because they were denied hospital visitation: ‘We had two incidents during the [COVID‐19] lockdown where mum falls—by taking her to hospital by ambulance. One hospital would not allow me to go in at all’ [G9_carer].

Staff recognised that a lack of information provision regarding PLWD affected their care performance: ‘It becomes challenging to understand what is happening. And it may take a couple of days to understand how to manage that thing' [G10_staff]. Staff identified the need to partner with carers in all aspects of care, for example: ‘PLWD's usual behaviour’ [G7_staff], ‘communication style’ [G3_staff], ‘discharge planning' [G11_staff], ‘medication list’ [G13_staff] and ‘advance directives’ [G3_ staff]. Moreover, staff described a lack of continuity of care in the current health and social care systems: ‘You've got to start from scratch with rapport and everything like that’ [IN 2_staff]. They embraced the idea of a facilitator to enhance care continuity: ‘So having some continuity for those transitions with a support worker who follows you through all of those changes from home to hospital, back home, to placement, palliative care’ [IN 2_staff].

Overall, Theme 3 revealed that there are three types of factors contributing to carer stress, as described in the conceptual framework: care recipient factors (i.e. hospitalisations), carer factors (i.e. lack of knowledge of respite care or lack of the self‐efficacy needed to obtain it) and service provider factors (i.e. exclusion of PLWD with changed behaviour from respite care, and lack of support for and partnership with carers during hospitalisation). Participants perceived that a designated iSupport facilitator could mitigate these stressors and advocate on behalf of PLWD for inclusive respite care services.

## 5 DISCUSSION

This study aimed to explore stakeholders' perceptions of a designated facilitator's role in supporting carers when embedding iSupport for Dementia programme into dementia care services in hospital and community aged care settings. Workshops and interviews were used to achieve the study's aim. Our findings confirm that care recipient factors, carer factors and service provider factors, as described in the study's conceptual framework (Figure 1), give rise to the multiple sources of stress experienced by carers. Moreover, carers face significant challenges in accessing and utilising social and health care services and mental health support to help them cope with these stressors.

Our findings revealed that participants were supportive of the facilitator role. They also stated that the facilitator role should be present (1) at the time of dementia diagnosis by helping carers access psychoeducation, information on care services and social support; (2) on a daily basis throughout the dementia care journey by acting as an educator and a resource person to enable carers to manage changed behaviour and medication and cope with emotional and psychological stress; and (3) during transition moments by advocating inclusive respite care services and supporting carers during the hospital stays of PLWD. Our findings also indicate that although carers' journeys have similar elements, their needs are not homogeneous. Thus, the iSupport facilitator will need to assess carers' needs for support and provide individualised support to address those needs. The findings of this study are discussed in detail in Sections 5.1, 5.3.

### 5.1 Supporting carers at the time of dementia diagnosis

Our study indicated that the time of receiving a diagnosis was difficult and stressful for carers as there was a lack of guidance and information regarding available services and planning strategies. This finding aligns with previous studies that highlighted an absence of detailed information on dementia and available support services (Tuijt et al., 2021). A recent Australian survey study by Mansfield et al. (2022) indicated that 97%–100% of geriatricians agreed that as part of the consultation at diagnosis, it was necessary to routinely provide information about disease progression, available support, and legal options for appointing substitute decision‐makers. However, participants in our study perceived that care service providers were unable to provide this information due to time constraints related to workload or the complexity of the aged care system. Concerns about information overload were also identified in our study, which supports the argument of Stokes et al. (2014) that highly emotional reactions to the diagnosis (i.e. stress, shock) may impact a carer's information retention ability. Therefore, a personalised approach to ongoing support for carers is more appropriate than a standardised one.

Unclear roles and responsibilities among health and social care professionals providing postdiagnosis support for carers of PLWD may cause confusion and result in information omission, leading to unnecessary carer stress. Mansfield et al. (2022) argued that some topics, such as advance directives, might be appropriate for GPs to initiate because the geriatricians who are responsible for dementia diagnosis in tertiary hospitals have no ongoing role in the care of PLWD. Our study suggests that having a facilitator as a single point of contact for carers, available during the process of diagnosis and for after‐diagnosis support, is highly regarded by stakeholders as a practical solution to these concerns.

### 5.2 Supporting carers through the everyday dementia care journey

Consistent with previous study results (Peeters et al., 2010), our study also found that lack of timely, ongoing, personalised carer support to manage changed behaviour at home caused significant stress for carers. Changed behaviour is highly prevalent; it affects up to 90% of PLWD at some stage (Burley et al., 2021). Carers in our study adopted various strategies to deal with changed behaviour; one example was modifying their communication style to reduce their negative thoughts about the changed behaviour of PLWD. These findings are consistent with those of a previous systematic review (Feast et al., 2016).

Health and social care professionals in our study also expressed the importance of fostering a partnership with carers to help them understand the causes of changed behaviour in PLWDs and stated that having an iSupport facilitator would be a way to strengthen the partnership. Trivedi et al. (2019) reported in their systematic review that trained professionals could improve carer competence in managing changed behaviours by focusing on problem‐solving techniques and coping strategies. Similar findings were reported by Stephan et al. (2018), who also recommended having a single‐point‐of‐contact person for carers to optimise support for them in preventing and managing changed behaviours of PLWD. This kind of support would alleviate the negative impact of care recipient factors on carers.

Furthermore, our study identified that carers were stressed about ongoing medication management, which was complicated by frequent medication changes, conflicting information, lack of knowledge about medication and lack of universal medication management support in the community. Our findings support those of a systematic review that indicated that carer stress was associated with medication management (Lim & Sharmeen, 2018). Our study indicates that carers are most likely to ask a community pharmacist their medication‐related questions to avoid conflicting information provided by different prescribers. Other studies suggested that GPs and pharmacists should work collaboratively in medication management in the community (Barry & Hughes, 2020). Dissatisfaction with GPs' and pharmacists' medication management was also described in our study and previously (El‐Saifi et al., 2021). Having a facilitator for carers could be a practical solution by facilitating collaborations between GPs and pharmacists. For example, GPs could use facilitators to support carers' ongoing medication management education. A facilitator could reinforce medication management advice provided by GPs and pharmacists or communicate with GPs or pharmacists to ensure medication reviews are timely and accurate information is provided to carers.

In our study, both carers and staff considered mental health support crucial. However, carers described being reluctant to seek support during emotional stress. Zwingmann et al. (2020) reported carers' rejection of mental health support, and found that the reasons were related to personal issues (i.e. time constraints), services issues (i.e. availability) and relational issues (i.e. preference). Our study added that a lack of mental health self‐care awareness and a lack of trust in health professionals also contributed to carers' apprehension about mental health support. Facilitators could assist health and social care professionals with building rapport with carers, refer carers to relevant mental health care providers and help them demystify mental health care.

### 5.3 Supporting carers during transition moments

Carers in our study detailed the difficulties they experienced when accessing respite care services. Our finding aligns with that of Shea et al. (2017), who highlighted poor respite service availability and accessibility. Our findings also support those of a previous study that demonstrated that carers showed a lack of knowledge of the available respite services (Phillipson et al., 2019). Carers in our study also reported that they had to use hospital beds to care for their loved ones when there were no adequate support services, such as respite in the community for carers. This finding reveals that a lack of respite care is associated with potentially avoidable burdens and costs for acute care hospitals. In Australia, in 2016 and 2017, about 50% of people hospitalised for PLWD were discharged home and 13% were awaiting residential aged care (AIHW, 2019), suggesting that the burdens of hospital stays (including the costs to the healthcare system) could be diminished by using facilitators.

### 5.4 Limitations

There are some limitations to our study. First, this study was conducted in Australia, which has a comprehensive health and social care system. The findings may not be transferable to other countries with different health and social care systems. Second, all our participants were fluent in English, and their views may not represent those of people in Australia with limited English proficiency. Third, despite our efforts, no carers of Aboriginal Australians participated in our study. Therefore, the results do not represent Australia's diverse population and need to be interpreted accordingly. Fourth, the use of online workshops and telephone interviews may have prevented the researchers from noticing participants' non‐verbal cues. Finally, some participants had cared for their loved ones for many years, which could have led to recall bias regarding distant events.

## 6 CONCLUSION

Caring for family members with dementia is mentally and physically demanding and stressful for carers and has a potentially adverse impact on their health and well‐being throughout their caring journeys. Our study confirms that the lack of support for carers in hospital settings and community aged care settings adds further sources of stress during a carer's journey. Our study identified that a facilitator‐enabled iSupport intervention in hospital and community aged care settings may mitigate sources of stress related to care recipient factors, carer factors and care service factors, thus potentially improving the health and well‐being of carers while addressing the care needs of PLWD. Moreover, our study also identified how the facilitator should be involved at the time of dementia diagnosis, in everyday dementia care and during transition moments, which will help hospitals and community aged care organisations develop new care services to support carers of PLWD.

## 7 RELEVANCE TO CLINICAL PRACTICE AND EDUCATION

To better support carers of PLWD in the community, health and social care professionals, care service providers and policymakers need to address all aspects of the sources of stress faced by carers. Our findings will inform the deployment of iSupport facilitators; their roles and responsibilities in delivering the planned interventions; and education and training for this group of health and social care professionals. Our findings are relevant to nurse‐led or coordinated dementia care in hospital and community aged care settings. First, the facilitator could collaborate with a geriatrician and a GP to ensure the carer is supported at the time of dementia diagnosis. For example, a GP or geriatrician could refer a newly diagnosed PLWD and their carer to the facilitator for dementia care education and for support in accessing resources. The facilitator could also assist PLWD and their carers to understand and navigate care services and identify those that are relevant to them. Second, the facilitator could provide ongoing carer needs assessment in addition to needs assessment for PLWD at the different dementia stages. This would allow health care and social care professionals to promptly address carers' challenges and improve the quality of home‐based dementia care. Third, as the facilitator would have a close working relationship with carers, they would be in an ideal position to advocate changes in practice and in care service development to meet the care needs of PLWD and their carers. Fourth, they could provide feedback on care services to facilitate continuous quality improvement in their organisation and be a resource person or mentor for their peers to enhance their team's approach to dementia care. Finally, further research is needed to investigate facilitator‐enabled iSupport interventions and evaluate the impact of the facilitator on carers, PLWD and the health care system (e.g. the cost of the health care) using randomised controlled trials. Qualitative studies investigating the experiences of PLWD, their carers and facilitators during facilitator‐enabled iSupport interventions are also needed.

## AUTHOR CONTRIBUTIONS

*Data collection*: Ying Yu, Sarah C. Hunter, Claudia Meyer, Michael Chapman and Kaiping Tan; *data analysis*: Ying Yu and Lily Xiao; draft of the manuscript: Ying Yu; development of original project idea and concepts: all authors; and final manuscript preparation for publication: all authors.

## FUNDING INFORMATION

This study is funded by the Australian Government via 2020 NHMRC/Medical Research Future Fund and NHMRC/The Dementia Centre for Research Collaboration (DCRC) World‐Class Research Project Grants.

## CONFLICT OF INTEREST STATEMENT

No potential competing interest was reported by the authors.

## ETHICS STATEMENT

Ethics approval was obtained from the Southern Adelaide Clinical Human Research Ethics Committee (No.2021/HRE00273) and Bolton Clarke Human Research Ethics Committee (No.21007).

## Supporting information


Data S1:

## Data Availability

Data available on request due to privacy/ethical restrictions.
